# Improvements in Gait Function Following Integrative Korean–Western Medicine Treatment in Older Adults with Chronic Stroke: A Retrospective Exploratory Study

**DOI:** 10.3390/medicina62091781

**Published:** 2026-09-16

**Authors:** Hyangyu Park, Yeonghun Wi, Cheol-Hyun Kim

**Affiliations:** 1 College of Korean Medicine, Wonkwang University, Iksan-daero 460, Iksan 54538, Jeonbuk-do, Republic of Korea; 62bless@naver.com (H.P.); wizwi@naver.com (Y.W.); 2 Department of Internal Medicine, College of Korean Medicine, Wonkwang University, Iksan-daero 460, Iksan 54538, Jeonbuk-do, Republic of Korea

**Keywords:** chronic stroke, gait disorders, gait symmetry, integrative Korean–Western medicine, older adults, rehabilitation

## Abstract

*Background and Objectives:* Patients with chronic stroke aged 65 years or older and who are more than 6 months post-onset are generally considered to have reached a rehabilitation plateau, beyond which further functional improvement is difficult to achieve. This study aimed to evaluate changes in gait function before and after integrative Korean–Western medicine treatment in older adults with chronic stroke. *Materials and Methods:* In this single-center, retrospective, single-group pre–post observational study, we analyzed the medical records of 15 patients with chronic stroke aged ≥65 years who received integrative Korean–Western medicine treatment five times per week for 4 weeks. The primary outcome was the step length ratio, reflecting gait symmetry. Secondary outcomes included walking velocity, lateral symmetry, center of pressure (COP)-related parameters, manual muscle test (MMT) scores, and Fugl-Meyer Assessment (FMA) scores. *Results:* The step length ratio increased significantly from 0.61 [0.50–0.70] before treatment to 0.86 [0.62–0.94] after treatment (*p* = 0.009, *r* = 0.675). Walking velocity (*p* = 0.006, *r* = 0.715) and mean COP velocity (*p* = 0.007, *d* = −0.822) also improved significantly. In contrast, no significant changes were observed in MMT or FMA scores, and no treatment-related adverse events were recorded. *Conclusions:* In this retrospective, uncontrolled, single-group study, significant improvements in gait symmetry, walking velocity, and postural control were observed following integrative Korean–Western medicine treatment in older adults with chronic stroke. The improvement in gait function without a corresponding change in FMA-based motor function—although both MMT and FMA scores were relatively high at baseline, potentially limiting sensitivity to detect further motor improvement—raises the hypothesis that other mechanisms, potentially including enhanced sensorimotor integration, may have contributed to these changes; however, this mechanistic interpretation remains speculative and was not directly tested. Randomized controlled trials with appropriate control groups are warranted to further verify these findings.

## 1. Introduction

Stroke is one of the most common causes of acquired disability in adulthood, and the number of stroke survivors continues to increase owing to population aging and advances in acute-phase care [1,2]. In particular, residual disability is common among older adults aged 65 years or older, making the maintenance and improvement of function beyond the acute phase a central goal of rehabilitation [3,4].

Among post-stroke sequelae, impaired gait function is a major factor directly affecting the ability to perform activities of daily living, fall risk, social participation, and quality of life [5]. Gait function is not merely a measure of mobility but also an indicator of whether independent living can be maintained. In older adults with stroke, gait impairment is closely associated with institutionalization and increased caregiver burden [6]. Therefore, exploring the potential for improving gait function even in older adults with chronic stroke has important clinical significance.

However, neurological recovery after stroke is known to occur largely within the first 3–6 months after onset, following which the rate and extent of spontaneous recovery decline markedly, making further functional gains difficult to achieve [7]. In older adults, further functional gains may be limited by reduced neuroplasticity, sarcopenia, and multimorbidity [8,9,10]. Consequently, older adults more than 6 months post-onset are often regarded as having reached a so-called rehabilitation plateau and may therefore be less likely to receive active treatment.

In clinical practice, however, cases of gait function change or improvement have been observed even beyond 6 months after stroke onset [11]. These observations suggest that changes in muscle strength and motor function do not necessarily parallel changes in gait function, and that gait improvement may occur through mechanisms other than strength recovery, such as enhanced sensorimotor integration or more efficient postural control [12,13,14].

Meanwhile, integrative Korean–Western medicine treatment (IKWMT) is widely used to manage post-stroke sequelae in Korean medicine hospitals [15,16]. IKWMT combines acupuncture and electroacupuncture with Western medical rehabilitation, including exercise and occupational therapy, and, in some patients, ultrasound-guided acupotomy. The specific components, frequencies, and durations used in this study are detailed in Section 2.3. Acupuncture has been proposed to stimulate afferent nerve fibers distributed in muscle spindles and Golgi tendon organs, potentially increasing proprioceptive sensory input and contributing to motor control and postural stability [17,18]. It may therefore act complementarily with rehabilitation therapy. However, most previous studies have targeted patients in the acute or subacute phase and have largely relied on clinical rating scales for assessment; studies confirming treatment responses through quantitative gait analysis in older adults with chronic stroke remain scarce.

Accordingly, this study aimed to retrospectively analyze changes in gait function, postural control, and neurological function before and after 4 weeks of IKWMT in patients with chronic stroke aged 65 years or older, who were more than 6 months post-onset. The primary outcome was the step length ratio, which reflects gait symmetry. In addition, by comparing the patterns of change in gait and postural control measures with those in muscle strength and motor function, we explored the potential mechanisms underlying gait changes in older adults with chronic stroke.

## 2. Materials and Methods

### 2.1. Study Aim and Design

This was a single-center, retrospective, exploratory observational study with a single-group pre–post design. Existing medical records from a single institution were retrospectively reviewed to evaluate changes in gait function before and after IKWMT in older adults with chronic stroke. We reviewed the medical records of patients with stroke who received care at Wonkwang University Gwangju Medical Center between 1 January 2024 and 31 May 2026, received IKWMT for 4 weeks, and underwent gait assessments before and after treatment. All patients had been receiving conventional rehabilitation therapy at other institutions and were admitted to our institution to receive this 4-week IKWMT protocol as part of routine clinical care; the medical records generated during this clinical treatment were subsequently reviewed retrospectively for the present study. No separate control group was established; within-subject changes were analyzed by comparing the pre- and post-treatment assessment results of each participant.

### 2.2. Participants

#### 2.2.1. Inclusion Criteria

Patients were included if they had been diagnosed with ischemic or hemorrhagic stroke based on brain imaging findings; were aged 65 years or older at the time of the first assessment; were at least 6 months post-onset of stroke at the time of the first assessment; received IKWMT for 4 weeks; underwent gait analysis and balance assessment both before and after treatment; and had pre- and post-treatment manual muscle test (MMT) and Fugl-Meyer assessment (FMA) results available in their medical records.

#### 2.2.2. Exclusion Criteria

Patients were excluded if they had been diagnosed with traumatic intracerebral hemorrhage or traumatic subarachnoid hemorrhage; had neurological disorders other than stroke or orthopedic conditions that could affect gait function; or could not undergo pre–post comparisons because of missing key assessment data.

#### 2.2.3. Sample Size Determination

**Figure 1:**
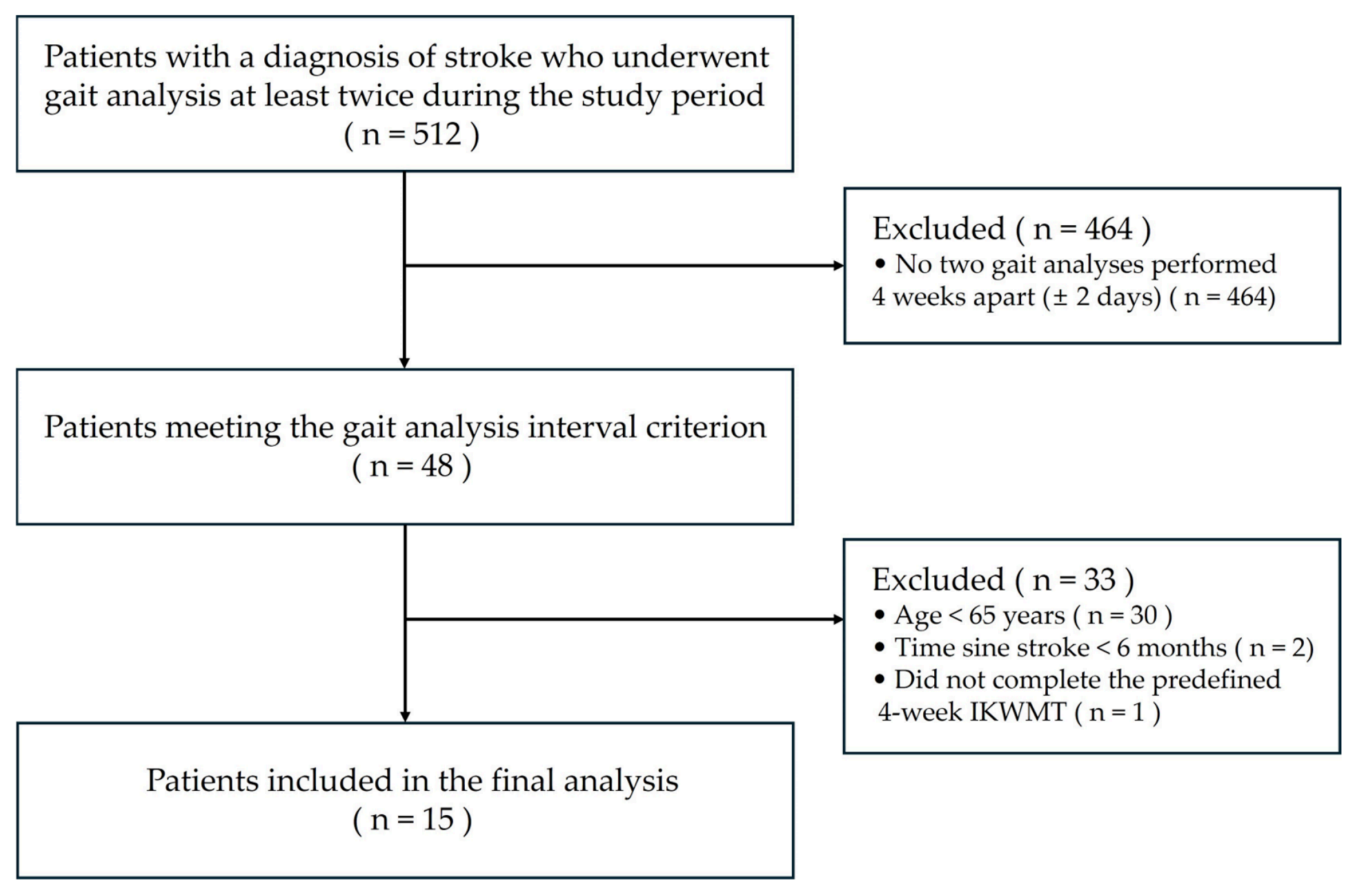
Flow diagram of participant screening and selection. The study period was from 1 January 2024 to 31 May 2026. Exclusion criteria were applied sequentially in the order shown (age, then time since stroke onset, then treatment completion), such that each patient was counted only under the first applicable exclusion criterion. IKWMT, integrative Korean–Western medicine treatment.

As this was a retrospective observational study, no a priori sample size calculation based on statistical power was performed. All eligible patients who met the inclusion criteria during the study period were included in the analysis. Medical records from the study period were screened to identify patients with a diagnosis of stroke who had undergone gait analysis at least twice. Among 512 patients identified, 48 had two gait analyses performed 4 weeks apart (±2 days). The predefined eligibility criteria described above were then applied sequentially to these 48 patients in the following order: age, time since stroke onset, and completion of the predefined 4-week IKWMT protocol. Patients meeting more than one exclusion criterion were categorized under the first criterion applied (Figure 1).

### 2.3. Interventions

All participants received IKWMT five times per week for 4 weeks. The integrative treatment consisted of acupuncture, electroacupuncture, and rehabilitation therapy. As common interventions, acupuncture needles were retained for 20 min per session, and electroacupuncture was applied at 4 Hz for 20 min. Exercise and occupational therapy were each administered for 30 min per session, focusing on strengthening and task-oriented functional training. Ultrasound-guided acupotomy was performed once weekly in patients with a Modified Ashworth Scale (MAS) score of 2 or higher at baseline, targeting the gastrocnemius, soleus, and tibialis posterior muscles under real-time ultrasound guidance. No other concomitant treatments were administered during the 4-week treatment period. Specifically, no additional rehabilitation therapy beyond the exercise and occupational therapy described above, pharmacological treatment for spasticity, botulinum toxin injections, or orthotic interventions were used during this period.

### 2.4. Outcome Measures and Assessments

All outcome data were obtained from assessments documented in the medical records before treatment initiation and after completion of the 4-week treatment as part of routine clinical care. Follow-up assessments after completion of treatment were not included in this study. All assessments were conducted as part of routine clinical practice, and assessor blinding was not applied owing to the retrospective nature of the study.

Gait and postural control were assessed using a treadmill-based gait analysis system with an integrated pressure platform (FDM-T; Zebris Medical GmbH, Isny, Germany). Assessments were performed without shoes. For gait assessment, participants walked for 30 s at a self-selected comfortable speed. For postural control assessment, participants maintained a static standing posture on the stationary treadmill for 10 s. Prior to the formal assessment, participants completed a treadmill walking practice trial of approximately 5 min to become familiar with treadmill walking, in line with previous research suggesting that treadmill gait familiarization occurs within several minutes in both older and younger adults [19]. Throughout both the gait and postural control assessments, participants walked and stood without holding the handrails, and a clinician remained standing beside the participant at all times to prevent falls. The same assessment protocol, including the use of the treadmill-based system, walking at a self-selected comfortable speed, and absence of handrail support, was maintained at both the pre- and post-treatment assessments, consistent with the treadmill-based gait assessment protocol used in our group’s previous studies [20].

The primary outcome was the step length ratio. Step length was defined as the anteroposterior distance from the heel contact point of one foot to the heel contact point of the contralateral foot. The step length ratio was calculated by dividing the step length of the paretic side by that of the non-paretic side, with values closer to 1 indicating greater symmetry between the two sides [21].

Secondary outcomes were categorized into the gait and postural control domain and the motor function domain. The gait and postural control domain included walking velocity, lateral symmetry, the 95% confidence ellipse area of the center of pressure (COP), and mean COP velocity. Walking velocity is an important indicator of walking performance in patients with stroke [21]. Lateral symmetry is a measure of the mediolateral deviation of the COP during gait; the absolute deviation from the midline was used for analysis regardless of the direction of deviation, with values closer to 0 indicating less mediolateral weight-shift asymmetry during gait [22].

The 95% confidence ellipse area of the COP was defined as the area of the ellipse encompassing 95% of the COP trajectory during static standing, and mean COP velocity was defined as the average speed of COP movement during static standing. For both variables, higher values indicate greater or faster postural sway and lower static postural stability [20].

The motor function domain included MMT and FMA of the upper and lower extremities. MMT results were obtained from assessments performed on a 0–5 scale, in which muscle strength is evaluated through examiner observation, palpation, and applied resistance, with higher scores indicating greater muscle strength [23]. The FMA is a standardized scale for assessing post-stroke motor function, with maximum scores of 66 for the upper extremity and 34 for the lower extremity; higher scores indicate better motor function [24].

Baseline clinical characteristics obtained from the medical records included age, sex, paretic side, stroke type, time since stroke onset, lower-limb spasticity assessed using MAS, and ambulatory function assessed using the Functional Ambulation Category (FAC). Lesion location, cortical versus subcortical involvement, and whether the stroke was a first-ever or recurrent event were determined from brain imaging reports in the medical record. For safety evaluation, the occurrence and details of adverse events documented in the medical records during the treatment period were also retrospectively reviewed. Adverse events were ascertained passively, based on symptoms spontaneously reported by patients during clinical visits and documented in the medical record; no standardized adverse event checklist or systematic active screening was used.

### 2.5. Statistical Analysis

The normality of the pre–post differences (after treatment minus before treatment) for each outcome variable was assessed using the Shapiro–Wilk test. When the differences satisfied the normality assumption (*p* ≥ 0.05), the paired *t*-test was applied; when normality was not satisfied (*p* < 0.05), the Wilcoxon signed-rank test was used.

Descriptive statistics are presented as the mean ± standard deviation for normally distributed variables and as the median with interquartile range (IQR) for non-normally distributed variables.

Effect sizes were calculated as rank-biserial correlation (r) for variables analyzed using the Wilcoxon signed-rank test and as Cohen’s *d* for variables analyzed using the paired *t*-test. Effect sizes were interpreted according to Cohen’s criteria as negligible (*r* < 0.1 or |*d*| < 0.2), small (*r* ≥ 0.1 or |*d*| ≥ 0.2), medium (*r* ≥ 0.3 or |*d*| ≥ 0.5), or large (*r* ≥ 0.5 or |*d*| ≥ 0.8) [25]. Additionally, 95% confidence intervals (CIs) were calculated for all effect-size estimates: for Cohen’s *d*, using the standard analytic approximation for paired-samples designs (SE = √(1/*n* + *d*^2^/(2(*n* − 1)))); for the rank-biserial correlation, using a percentile bootstrap procedure with 10,000 resamples of the paired differences, given the absence of a simple closed-form CI for this statistic.

All hypothesis tests were two-sided, with the significance level set at α = 0.05. Because this was an exploratory study in which the step length ratio was prespecified as the primary outcome, no correction for multiple comparisons was performed, and the results for secondary outcomes were interpreted as exploratory and hypothesis-generating, including outcomes that reached statistical significance. All statistical analyses were performed using IBM SPSS Statistics for Windows, Version 23.0 (IBM Corp., Armonk, NY, USA). An exploratory Spearman’s rank correlation analysis was additionally performed to examine the association between time since stroke and the change in step length ratio (ΔSLR, calculated as the post-treatment minus the pre-treatment step length ratio for each patient); given the small sample size and the considerable heterogeneity in time since stroke, this analysis was regarded as hypothesis-generating rather than confirmatory.

## 3. Results

### 3.1. Participant Selection

A total of 512 patients with a diagnosis of stroke who underwent gait analysis at least twice during the study period were identified. Of these, 464 were excluded because they did not have two gait analyses performed 4 weeks apart (±2 days), leaving 48 patients. Among these 48 patients, the predefined eligibility criteria were then applied sequentially: 30 were excluded because they were younger than 65 years, two because the time since stroke onset was less than 6 months, and one because the predefined 4-week IKWMT protocol was not completed. Consequently, 15 patients were included in the final analysis (Figure 1).

### 3.2. Baseline Characteristics of Participants

A total of 15 patients were included in the final analysis (Table 1). The mean age was 70.7 ± 4.3 years (range, 65–78 years), and the median time since stroke onset was 42 [21–123] months, with a range of 6–256 months. Six patients (40.0%) were male and nine (60.0%) were female. The paretic side was the right in nine patients (60.0%) and the left in six (40.0%), and the stroke type was ischemic in eight patients (53.3%) and hemorrhagic in seven (46.7%). Regarding lesion characteristics, the most common lesion site was the basal ganglia (*n* = 6), followed by the internal capsule (*n* = 2) and pons (*n* = 2); the remaining patients had lesions in the thalamus (*n* = 1), the frontal cortex alone (*n* = 1), both the cortical and subcortical regions (*n* = 2, including one large middle cerebral artery territory infarct involving the frontoparietal cortex, basal ganglia, and internal capsule), or a non-parenchymal subarachnoid hemorrhage (*n* = 1). Overall, lesions were subcortical only in 9 patients (60.0%), cortical and subcortical in 2 (13.3%), cortical only in 1 (6.7%), infratentorial in 2 (13.3%), and non-parenchymal in 1 (6.7%); 14 patients (93.3%) had a first-ever stroke and 1 (6.7%) had a recurrent stroke (Table 1).

The median MAS score for lower-limb spasticity was 2 [1–2], and eight patients (53.3%) had an MAS score of 2 or higher. The median FAC score, reflecting ambulatory function, was 3 [3–4]; eight patients (53.3%) had an FAC score of 3 and seven (46.7%) had a score of 4.

### 3.3. Characteristics of IKWMT

The components of IKWMT administered to the participants are presented in Table 2. All participants received acupuncture, electroacupuncture, exercise therapy, and occupational therapy five times per week for 4 weeks. Eight patients (53.3%) with a baseline MAS score ≥ 2 for lower-limb spasticity additionally received ultrasound-guided acupotomy once weekly.

**Table 2:** Components of integrative Korean–Western medicine treatment.

Treatment	Recipient	Frequency/Duration	Description
Acupuncture	All patients (*n* = 15)	Daily (5 days/week)	Sterile disposable needles inserted at standardized acupoints for the lower extremities and trunk; retained for 20 min per session.
Electroacupuncture	All patients (*n* = 15)	Daily (5 days/week)	Electrical stimulation (4 Hz) applied via needles at selected acupoints for 20 min per session.
Exercise therapy	All patients (*n* = 15)	Daily (5 days/week)	Neurodevelopmental treatment-based physiotherapy including gait training, balance training, and strengthening exercises; 30 min/session.
Occupational therapy	All patients (*n* = 15)	Daily (5 days/week)	Upper extremity functional training, activities of daily living practice, and fine motor skill exercises; 30 min/session.
Ultrasound-guided acupotomy	MAS ≥ 2 (*n* = 8)	Once per week	Thread-blade needle inserted under real-time ultrasound guidance targeting spastic muscles of the lower extremities (gastrocnemius, soleus, and tibialis posterior muscles).

All treatments were administered over a 4-week inpatient period. Ultrasound-guided acupotomy was selectively applied to patients with a baseline MAS score ≥ 2 for lower-limb spasticity as an adjunct to the standard integrative treatment protocol. MAS, Modified Ashworth Scale.

### 3.4. Primary Outcome: Step Length Ratio

The step length ratio (primary outcome) increased significantly from 0.61 [0.50–0.70] before treatment to 0.86 [0.62–0.94] after treatment (Wilcoxon signed-rank test, *p* = 0.009), with a large effect size (*r* = 0.675, 95% CI 0.280–0.883) (Table 3).

To clarify the biomechanical source of this improvement, the paretic- and non-paretic-side step lengths were examined separately. Paretic-side step length increased significantly from a median of 8.00 [5.00–11.50] cm before treatment to 13.00 [6.50–18.00] cm after treatment (Wilcoxon signed-rank test, *p* = 0.033), with a large effect size (*r* = 0.552, 95% CI 0.123–0.854). In contrast, non-paretic-side step length did not change significantly, from a median of 16.00 [11.00–18.00] cm before treatment to 15.00 [13.00–20.00] cm after treatment (*p* = 0.479), with a small effect size (*r* = 0.183, 95% CI 0.000–0.652) (Table 3). These findings suggest that the improvement in step length ratio primarily reflected an increase in paretic-side step length, rather than a change in the non-paretic side.

**Figure 2:**
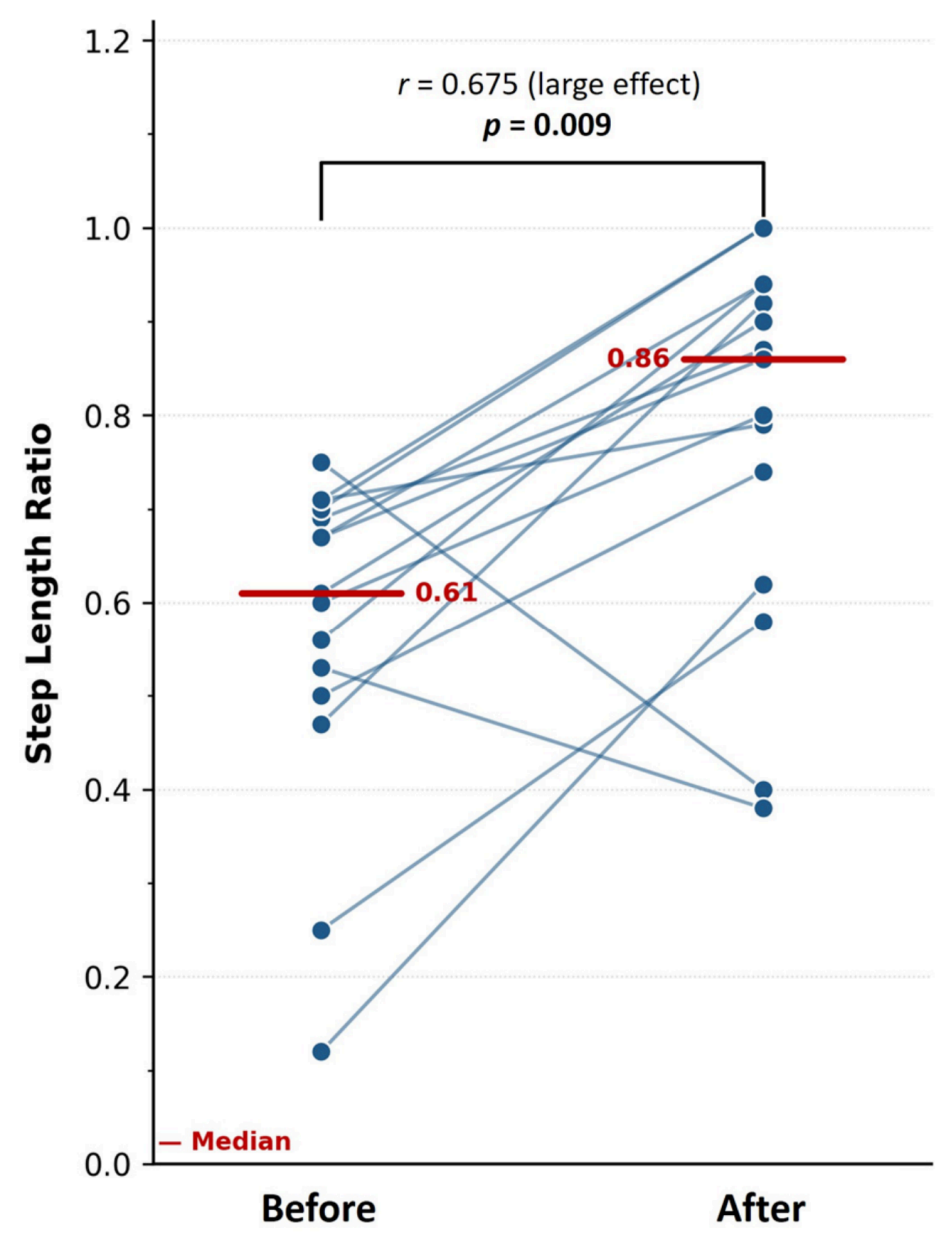
Individual changes in step length ratio before and after integrative Korean–Western medicine treatment (*n* = 15). Each dot represents an individual patient, and connecting lines indicate individual changes from before to after treatment. Of the 15 patients, the step length ratio increased in 13 and decreased in 2. Horizontal bars indicate the group median. The dotted horizontal line at 1.0 indicates perfect gait symmetry. Statistical significance was assessed using the Wilcoxon signed-rank test. *r*, rank-biserial correlation (effect size).

The individual patterns of change before and after treatment are presented in Figure 2. Of the 15 patients, the step length ratio increased in 13 and decreased in 2 (0.75 → 0.40 and 0.53 → 0.38). Among the patients who improved, the two patients with the lowest pre-treatment step length ratios increased from 0.12 to 0.62 and from 0.25 to 0.58, respectively, while another two patients reached a step length ratio of 1.00 after treatment.

To explore whether this heterogeneity in time since stroke was associated with the magnitude of change in step length ratio (ΔSLR), an exploratory Spearman correlation analysis was performed between time since stroke and ΔSLR for each patient. No statistically significant association was observed (Spearman’s ρ = 0.043, *p* = 0.879); given the small sample size, however, this exploratory analysis was underpowered, and the near-zero correlation coefficient should not be interpreted as definitive evidence of no association. Individual data points are presented in Figure S1.

The 95% confidence intervals (CIs) for Cohen’s *d* were computed using the standard analytic approximation for paired-samples effect sizes; 95% CIs for the rank-biserial correlation (*r*) were estimated using a percentile bootstrap procedure (10,000 resamples of the paired differences). For walking velocity and upper-extremity FMA, the bootstrap-derived point estimates of *r* (0.697 and 0.390, respectively) differed slightly (≤0.02) from the *r* values originally computed using IBM SPSS Statistics and reported in this table; this reflects minor differences in the handling of tied values between statistical software packages rather than a data inconsistency, and the originally reported point estimates have been retained.

For MMT-Upper extremity, all 15 patients had identical scores before and after treatment; because there was no variability in the paired differences, a 95% CI could not be estimated. For FMA-Lower extremity, 11 of 15 patients showed no change and the remaining 4 patients all showed an increase (i.e., no patient’s score decreased); this consistent direction of change, despite involving few patients, accounts for a bootstrap CI that does not include zero even though the Wilcoxon signed-rank test itself was not statistically significant (*p* = 0.068).

### 3.5. Secondary Outcomes: Gait and Postural Control

Walking velocity increased significantly from 0.40 [0.30–0.50] km/h before treatment to 0.50 [0.40–0.80] km/h after treatment (*p* = 0.006), with a large effect size (*r* = 0.715, 95% CI 0.459–0.838) (Table 3).

Lateral symmetry decreased from 15.00 ± 9.86 mm before treatment to 13.77 ± 15.31 mm after treatment, but the difference was not statistically significant (*p* = 0.666), with a negligible effect size (*d* = −0.114, 95% CI −0.669 to 0.442).

The 95% confidence ellipse area of the COP decreased from 781.1 ± 662.6 mm^2^ before treatment to 373.9 ± 373.5 mm^2^ after treatment, but this change did not reach statistical significance (*p* = 0.057), with a medium effect size (*d* = −0.536, 95% CI −1.131 to 0.058). Mean COP velocity decreased significantly from 18.87 ± 6.24 mm/s before treatment to 14.33 ± 5.18 mm/s after treatment (*p* = 0.007), with a large effect size (*d* = −0.822, 95% CI −1.468 to −0.176).

### 3.6. Secondary Outcomes: Muscle Strength and Motor Function

No statistically significant pre-post changes were observed in the muscle strength or motor function measures (Table 3). MMT scores of the upper extremity were 4.00 [3.00–4.00] both before and after treatment (*p* = 1.000, *r* = 0.000, 95% CI not estimable due to lack of variability), and MMT scores of the lower extremity likewise remained 4.00 [4.00–4.00] before and after treatment, showing no significant difference (*p* = 0.317, *r* = 0.258, small, 95% CI 0.000–0.447).

FMA scores of the upper extremity changed from 63.00 [38.00–66.00] before treatment to 63.00 [41.00–66.00] after treatment without statistical significance (*p* = 0.116, *r* = 0.406, medium, 95% CI 0.000–0.627), and FMA scores of the lower extremity increased from 28.00 [22.00–32.00] to 30.00 [22.00–32.00], but the change was not statistically significant (*p* = 0.068, *r* = 0.471, medium, 95% CI 0.258–0.634).

### 3.7. Adverse Events

Review of the medical records identified no adverse events related to IKWMT during the treatment period. As adverse events were ascertained passively, based on symptoms spontaneously reported by patients rather than through systematic active screening, this finding should be interpreted with caution and may underestimate the true incidence of mild or transient adverse events.

## 4. Discussion

In this study, IKWMT was administered to patients with chronic stroke aged 65 years or older who were at least 6 months post-onset of stroke, and changes in gait and neurological function before and after treatment were analyzed. The primary outcome, the step length ratio, improved significantly from 0.61 [0.50–0.70] before treatment to 0.86 [0.62–0.94] after treatment (*p* = 0.009, *r* = 0.675). The secondary outcome of walking velocity also improved significantly, from 0.40 [0.30–0.50] km/h to 0.50 [0.40–0.80] km/h (*p* = 0.006, *r* = 0.715). Mean COP velocity, reflecting postural control, likewise decreased significantly from 18.87 ± 6.24 mm/s to 14.33 ± 5.18 mm/s (*p* = 0.007, *d* = −0.822). All three variables showed large effect sizes, indicating that these changes were statistically significant and of large magnitude; whether they also meet established thresholds for clinical importance is discussed further below. .

These findings are particularly noteworthy in light of the characteristics of the study population. In older adults, further functional gains may be limited by reduced neuroplasticity, sarcopenia, and multimorbidity [26]. Moreover, beyond 6 months after stroke onset, the rate and extent of spontaneous neurological recovery are substantially reduced, making further functional improvement more difficult to achieve. Consistent with this, numerous studies in patients with chronic stroke have reported that the effects of rehabilitation are more limited in later phases than those observed in the subacute phase [27]. Against this background, the improvements in gait symmetry, walking velocity, and postural control observed following IKWMT in this study are of clinical interest.

Despite the significant improvements in gait function, the secondary outcomes assessing neurological function showed no statistically significant changes. For MMT, which assesses upper- and lower-limb muscle strength, no significant pre–post differences were observed for either the upper (*p* = 1.000) or lower extremity (*p* = 0.317). Notably, lower-extremity MMT scores were tightly clustered at the upper end of the 0–5 scale both before and after treatment (median, 4.00 [4.00–4.00]), leaving limited room to detect small changes in strength; therefore, the absence of a statistically significant change in MMT does not necessarily indicate an absence of small improvements in muscle strength. Similarly, for the FMA, which comprehensively assesses upper- and lower-limb motor function, neither the upper (*p* = 0.116) nor lower extremity (*p* = 0.068) showed a statistically significant change. Baseline FMA scores were also relatively high in this cohort (median upper-extremity FMA, 63 of a maximum 66; median lower-extremity FMA, 28 of a maximum 34), raising the possibility that reduced sensitivity related to high baseline scores, similar to that seen for MMT, may have also limited the ability to detect further improvement on the FMA. However, unlike lower-extremity MMT scores, which were tightly clustered at the ceiling (IQR, 4.00–4.00), the interquartile ranges for FMA were considerably wider (38–66 for the upper extremity and 22–32 for the lower extremity), indicating that baseline motor function was not uniformly at ceiling across participants; a ceiling-related reduction in sensitivity therefore remains possible for FMA, though it is less clearly demonstrated than for MMT. Nevertheless, the lower-extremity FMA showed a medium effect size (*r* = 0.471, 95% CI 0.258–0.634); as this result did not reach statistical significance, it should be interpreted as an exploratory, non-significant finding rather than a trend toward improvement, and a contribution of lower-extremity motor function to the observed gait changes cannot be confirmed by the present data. This interval does not include zero even though the corresponding Wilcoxon signed-rank test was not statistically significant; as detailed in the Table 3 footnote, this reflects the fact that 11 of the 15 patients showed no change at all in lower-extremity FMA and the remaining 4 all showed an increase, so the direction of any observed change was consistently positive despite the small number of patients who changed. Overall, the pattern of improved gait function without a significant change in FMA-based motor function suggests that the observed gait changes may not be fully explained by changes in motor function as measured by the FMA. Nonetheless, considering the relatively high baseline motor scores and the limited sensitivity of MMT—and, to a lesser extent, FMA—to detect small changes as noted above, a contribution from subtle motor or strength gains not fully captured by these measures remains possible.

It should be noted that neither proprioception nor sensorimotor integration was directly measured in this study; the following mechanistic interpretations are therefore presented as hypotheses generated from the observed pattern of results, rather than as explanations directly supported by the data. One possible mechanism is enhanced proprioceptive input associated with acupuncture. Proprioception conveys sensory information regarding joint position and the direction and velocity of movement to the central nervous system and is essential for maintaining a stable gait pattern. Acupuncture has been proposed to stimulate Aβ and Aδ afferent nerve fibers associated with muscle spindles and Golgi tendon organs, thereby increasing proprioceptive afferent input and improving motor control and postural stability [28]. Indeed, improvements in gait symmetry and balance following acupuncture have been reported in patients with stroke [18], supporting the possibility that gait patterns may improve through enhanced sensorimotor integration even in the absence of substantial changes in muscle strength. In particular, the improvement in step length ratio primarily reflected an increase in paretic-side step length, with no significant change on the non-paretic side; while this pattern is compatible with improved paretic-limb function rather than a compensatory strategy on the non-paretic side, it does not, on its own, confirm the proprioception-based mechanism proposed above.

This interpretation is also consistent with the changes in postural control measures. Mean COP velocity decreased significantly after treatment (*p* = 0.007, *d* = −0.822; large effect), suggesting reduced postural sway and improved postural control [29]. The 95% confidence ellipse area of the COP also decreased by approximately 52%, although this change was not statistically significant (*p* = 0.057, *d* = −0.536; medium effect) and should therefore be regarded as hypothesis-generating rather than evidence of a genuine trend. Taken together, these findings are compatible with, but do not provide direct evidence for, the proprioception-based mechanism proposed above.

In contrast, lateral symmetry showed no significant change after treatment (*p* = 0.666). In a previous study by our group, increased lateral weight shift toward the non-paretic side was observed in patients with stroke whose gait function had improved, suggesting a compensatory strategy to maintain dynamic balance as walking capacity increases [20,29,30]. Thus, the lack of significant change in lateral symmetry in the present study may reflect a similar compensatory reliance on the non-paretic limb, although this mechanism was not directly assessed.

IKWMT in this study combined Western medical rehabilitation with acupuncture-based interventions, including acupuncture, electroacupuncture, and, in selected patients, ultrasound-guided acupotomy. Whereas Western medical rehabilitation focuses on motor learning through strengthening and functional task training [3,4], acupuncture may provide complementary sensory input that could facilitate sensorimotor integration [31]. The observed improvements following IKWMT suggest that further functional gains may be possible even in older adults with chronic stroke.

This study has several limitations. First, the single-group pre–post design without a control group precludes causal attribution of the observed changes to IKWMT and cannot exclude the effects of time, natural recovery, regression to the mean, measurement variability, or changes in rehabilitation exposure. In particular, although all patients had previously received conventional rehabilitation at other institutions, the intensity and composition of prior rehabilitation were not systematically recorded. Second, the small sample size (*n* = 15) limits the generalizability and statistical power of the findings. In addition, effect-size estimates derived from such a small exploratory sample may be unstable and potentially overestimated and should therefore be interpreted cautiously until confirmed in larger prospective studies. Moreover, because multiple secondary outcomes were analyzed without correction for multiple comparisons, the possibility of Type I error cannot be excluded, and statistically significant secondary findings should therefore be regarded as exploratory and hypothesis-generating rather than confirmatory. Third, ultrasound-guided acupotomy was additionally administered to eight patients with lower-limb spasticity (MAS ≥ 2), resulting in a non-uniform treatment protocol. Because acupotomy may independently affect gait function through the release of fascial adhesions and reduction in spasticity [32], its contribution to the observed changes cannot be distinguished from that of the other treatment components. Descriptive outcomes according to acupotomy status are provided in Tables S1 and S2; however, the small, non-randomized subgroups preclude meaningful between-group inference. Future studies should standardize the treatment protocol or evaluate acupotomy as an independent treatment component. Fourth, no follow-up assessment was performed after treatment completion; therefore, the persistence of the observed improvements could not be evaluated. Fifth, because of the retrospective design, assessor blinding was not implemented, and treatment details were adjusted according to clinical judgment, introducing potential performance and measurement bias. Sixth, although significant changes with large effect sizes were observed in step length ratio, walking velocity, and mean COP velocity, their clinical importance remains uncertain. The observed increase in walking velocity was smaller than published minimal clinically important difference (MCID) and minimal detectable change (MDC) thresholds derived primarily from overground gait assessments in different stroke populations [33,34,35], and validated thresholds applicable to the treadmill-based gait speed, step length ratio, and COP velocity measures used in this study were unavailable. Moreover, although walking velocity increased from 0.40 to 0.50 km/h, the post-treatment walking speed remained well below gait-speed levels generally associated with independent community ambulation in individuals with stroke [36]. Thus, despite the statistically significant improvement, substantial functional limitations in walking capacity likely remained after treatment, and the findings should not be interpreted as evidence of established clinical meaningfulness. Accordingly, these findings should be interpreted as statistically significant changes rather than evidence of established clinical meaningfulness. Seventh, time since stroke onset varied considerably (6–256 months), and the exploratory analysis did not demonstrate a statistically significant association between time since stroke and change in step length ratio; however, the small sample size limits this analysis, and residual confounding remains possible. Eighth, adverse events were identified passively from spontaneously reported symptoms documented in medical records rather than through systematic active surveillance, potentially underestimating mild or transient events. Ninth, learning or familiarization effects from repeated assessments cannot be excluded. Although identical treadmill familiarization was performed before both gait assessments, no specific familiarization procedure was used for the static balance assessment. Tenth, because walking velocity was measured at a self-selected rather than fixed speed, the observed increase may partly reflect changes in confidence or willingness to walk faster rather than physiological improvement alone.

Despite these limitations, this study has several important clinical implications. First, it targeted older adults with chronic stroke who were at least 6 months post-onset, a population in whom spontaneous neurological recovery is relatively limited compared with the early recovery phase. Second, gait symmetry, walking velocity, and postural control were objectively evaluated using quantitative gait analysis in addition to clinical scales. Third, the findings suggest that further improvement in gait function may be possible in this population following multimodal rehabilitative interventions incorporating integrative Korean–Western medicine.

## 5. Conclusions

Despite the limitations of a single-group pre–post design and small sample size, this study found that IKWMT was associated with statistically significant improvements in gait symmetry, walking velocity, and some measures of postural control, with large effect sizes, in patients with chronic stroke aged 65 years or older who were at least 6 months post-onset. Future large-scale randomized controlled trials with appropriate control groups are needed to verify these findings, determine the persistence of the observed changes, and elucidate the independent contributions of each component of the intervention.

## Figures and Tables

**Table 1:** Baseline characteristics of participants (*n* = 15).

Characteristic	Value	*n* (%)
Age (years), mean ± SD	70.7 ± 4.3	–
Range	65–78	–
Stroke duration (months), median [IQR]	42 [21–123]	–
Range	6–256	–
Sex		
Male	–	6 (40.0)
Female	–	9 (60.0)
Paretic side		
Right	–	9 (60.0)
Left	–	6 (40.0)
Stroke type		
Ischemic	–	8 (53.3)
Hemorrhagic	–	7 (46.7)
Modified Ashworth Scale (MAS), median [IQR]	2 [1–2]	–
MAS 0	–	2 (13.3)
MAS 1	–	5 (33.3)
MAS 2	–	5 (33.3)
MAS 3	–	3 (20.0)
MAS ≥ 2 (received acupotomy)	–	8 (53.3)
Functional Ambulation Category (FAC), median [IQR]	3 [3–4]	–
FAC 3	–	8 (53.3)
FAC 4	–	7 (46.7)
Cortical/subcortical involvement		
Subcortical only	–	9 (60.0)
Cortical and subcortical	–	2 (13.3)
Cortical only	–	1 (6.7)
Infratentorial	–	2 (13.3)
Non-parenchymal (subarachnoid hemorrhage)	–	1 (6.7)
Stroke recurrence		
First-ever	–	14 (93.3)
Recurrent	–	1 (6.7)
Lesion location		
Basal ganglia	–	6 (40.0)
Internal capsule	–	2 (13.3)
Pons	–	2 (13.3)
Thalamus	–	1 (6.7)
Frontal cortex	–	1 (6.7)
Frontal cortex and basal ganglia	–	1 (6.7)
MCA territory involving the frontoparietal cortex, basal ganglia, and internal capsule	–	1 (6.7)
Subarachnoid space (non-parenchymal)	–	1 (6.7)

MAS was assessed for lower-limb spasticity. Patients with a baseline MAS score ≥ 2 additionally received ultrasound-guided acupotomy targeting lower-limb spastic muscles. FAC 3 = dependent ambulation on level surfaces; FAC 4 = independent ambulation on level surfaces. IQR, interquartile range; MAS, Modified Ashworth Scale; FAC, Functional Ambulation Category.

**Table 3:** Comparison of gait and motor functions before and after integrative Korean–Western medicine treatment (*n* = 15, age ≥ 65 years, onset ≥ 6 months).

Variable	Before (Mean ± SD or Median [IQR])	After (Mean ± SD or Median [IQR])	Effect Size (95% CI)	*p*-Value
Primary outcome				
Step length ratio	0.61 [0.50–0.70]	0.86 [0.62–0.94]	*r* = 0.675 (large) (95% CI 0.280–0.883)	0.009 *
Paretic	8.00 [5.00–11.50]	13.00 [6.50–18.00]	*r* = 0.552 (large) (95% CI 0.123–0.854)	0.033 *
step length (cm)				
Non-paretic	16.00 [11.00–18.00]	15.00 [13.00–20.00]	*r* = 0.183 (small) (95% CI 0.000–0.652)	0.479
step length (cm)				
Secondary outcomes				
Walking velocity (km/h)	0.40 [0.30–0.50]	0.50 [0.40–0.80]	*r* = 0.715 (large) (95% CI 0.459–0.838)	0.006 *
Lateral symmetry (mm)	15.00 ± 9.86	13.77 ± 15.31	*d* = −0.114 (negligible) (95% CI −0.669 to 0.442)	0.666
COP area (mm^2^)	781.1 ± 662.6	373.9 ± 373.5	*d* = −0.536 (medium) (95% CI −1.131 to 0.058)	0.057
COP velocity (mm/s)	18.87 ± 6.24	14.33 ± 5.18	*d* = −0.822 (large) (95% CI −1.468 to −0.176)	0.007 *
MMT-Upper extremity	4.00 [3.00–4.00]	4.00 [3.00–4.00]	*r* = 0.000 (negligible) (95% CI not estimable due to lack of variability)	1.000
MMT-Lower extremity	4.00 [4.00–4.00]	4.00 [4.00–4.00]	*r* = 0.258 (small) (95% CI 0.000–0.447)	0.317
FMA-Upper extremity	63.00 [38.00–66.00]	63.00 [41.00–66.00]	*r* = 0.406 (medium) (95% CI 0.000–0.627)	0.116
FMA-Lower extremity	28.00 [22.00–32.00]	30.00 [22.00–32.00]	*r* = 0.471 (medium) (95% CI 0.258–0.634)	0.068

Step length ratio: ratio of paretic to non-paretic step length (1.0 = perfect symmetry). Data are presented as median [IQR] for non-normally distributed variables (Wilcoxon signed-rank test) and mean ± SD for normally distributed variables (paired *t*-test). Normality was assessed using the Shapiro–Wilk test. Effect size: rank-biserial correlation (*r*) for Wilcoxon; Cohen’s *d* for paired *t*-test. Magnitude: negligible (*r* < 0.1 or |*d*| < 0.2), small (*r* ≥ 0.1 or |*d*| ≥ 0.2), medium (*r* ≥ 0.3 or |*d*| ≥ 0.5), and large (*r* ≥ 0.5 or |*d*| ≥ 0.8). CI, confidence interval; COP, center of pressure; COP area, 95% confidence ellipse area of the COP; COP velocity, mean velocity of the COP; FMA, Fugl-Meyer Assessment; MMT, manual muscle test. * indicates statistical significance (*p* < 0.05).

## Data Availability

The data presented in this study are available on request from the corresponding author.

## Supplementary Materials

The following supporting information can be downloaded at: https://www.mdpi.com/article/10.3390/medicina62091781/s1, Table S1: Gait and postural control outcomes before and after treatment, according to acupotomy status-; Table S2: Muscle strength and motor function outcomes before and after treatment, according to acupotomy status; Figure S1: Association between time since stroke and change in step length ratio (ΔSLR).

## Abbreviations

The following abbreviations are used in this manuscript:

CI	Confidence Interval
COP	Center of Pressure
FAC	Functional Ambulation Category
FMA	Fugl-Meyer Assessment
IKWMT	Integrative Korean–Western Medicine Treatment
IQR	Interquartile Range
MAS	Modified Ashworth Scale
MCID	Minimal Clinically Important Difference
MDC	Minimal Detectable Change
MMT	Manual Muscle Test
SD	Standard Deviation
ΔSLR	Change in Step Length Ratio
