# Supplementary material for: Improvements in Gait Function Following Integrative Korean–Western Medicine Treatment in Older Adults with Chronic Stroke: A Retrospective Exploratory Study
Source: Medicina (Kaunas). 2026 Sep 16;62(9):1781. doi: 10.3390/medicina62091781 (PMC13609465; doi:10.3390/medicina62091781)
Supplement: Supplementary file 1 [file medicina-62-01781-s001.zip › medicina-4560253-supplementary.pdf]

Supplementary Materials

Table S1. Gait and postural control outcomes before and after treatment, according to acupotomy status.

Acupotomy	Step length ratio		Walking velocity, km/h		Lateral symmetry, mm		COP area, mm²		COP velocity, mm/s	
	Before	After	Before	After	Before	After	Before	After	Before	After
Yes	0.47	0.92	0.3	0.7	36.0	25.4	577.0	174.0	15.0	18.0
Yes	0.61	0.90	0.5	0.9	10.6	5.9	1095.0	456.0	16.0	19.0
Yes	0.75	0.40	0.3	0.3	12.5	2.4	880.0	1538.0	21.0	22.0
Yes	0.53	0.38	0.2	0.3	23.6	48.2	606.0	289.0	25.0	14.0
Yes	0.60	0.80	0.4	0.4	23.0	40.0	246.0	846.0	22.0	20.0
Yes	0.12	0.62	0.3	0.5	26.0	12.1	1631.0	137.0	17.0	10.0
Yes	0.25	0.58	0.4	0.5	20.9	28.1	111.0	193.0	14.0	12.0
Yes	0.71	1.00	0.4	0.4	1.7	4.8	366.0	210.0	13.0	7.0
No	0.69	0.87	0.6	0.7	14.1	3.4	862.0	208.0	13.0	8.0
No	0.71	0.79	0.6	0.5	21.2	19.8	2665.0	270.0	35.0	16.0
No	0.67	0.94	0.5	0.9	11.1	0.4	257.0	111.0	12.0	9.0
No	0.67	0.86	0.3	0.3	11.4	12.1	743.0	500.0	24.0	21.0
No	0.56	0.94	0.4	0.8	3.7	0.4	349.0	242.0	19.0	13.0
No	0.70	1.00	0.5	0.8	2.1	1.4	283.0	130.0	14.0	8.0
No	0.50	0.74	0.4	0.5	7.1	2.2	1046.0	304.0	23.0	18.0
Yes, summary (n=8)	*0.56 [0.41–0.64]*	*0.71 [0.53–0.91]*	*0.3 [0.3–0.4]*	*0.5 [0.4–0.6]*	*19.3 ± 10.6*	*20.9 ± 17.3*	*689.0 ± 499.0*	*480.4 ± 486.5*	*17.9 ± 4.3*	*15.2 ± 5.3*
No, summary (n=7)	*0.67 [0.61–0.69]*	*0.87 [0.82–0.94]*	*0.5 [0.4–0.6]*	*0.7 [0.5–0.8]*	*10.1 ± 6.5*	*5.7 ± 7.4*	*886.4 ± 842.5*	*252.1 ± 130.0*	*20.0 ± 8.2*	*13.3 ± 5.2*

MAS, Modified Ashworth Scale (lower limb); COP, center of pressure. 'Acupotomy: Yes' (MAS ≥ 2, n = 8) denotes patients who additionally received ultrasound-guided acupotomy once weekly; 'Acupotomy: No' (MAS < 2, n = 7) denotes patients who received acupuncture, electroacupuncture, and rehabilitation therapy only. Individual rows list each patient's value before and after treatment; summary rows report median [IQR] (step length ratio, walking velocity) or mean ± SD (lateral symmetry, COP area, COP velocity) for each subgroup, consistent with the distributional classification used for the same variables in Table 3 for the full cohort (n = 15). Because acupotomy was administered according to baseline spasticity severity (MAS ≥ 2), the subgroups differed systematically at baseline and were not directly comparable. Therefore, these data are presented for descriptive purposes only and should not be interpreted as evidence of the independent effect of acupotomy; no formal between-group statistical testing was performed.

Table S2. Muscle strength and motor function outcomes before and after treatment, according to acupotomy status.

Acupotomy	MMT–Upper extremity		MMT–Lower extremity		FMA–Upper extremity		FMA–Lower extremity	
	Before	After	Before	After	Before	After	Before	After
Yes	3.00	3.00	4.00	4.00	29.00	30.00	15.00	15.00
Yes	4.00	4.00	4.00	4.00	63.00	62.00	28.00	32.00
Yes	2.00	2.00	3.00	3.00	28.00	28.00	22.00	22.00
Yes	3.00	3.00	3.00	3.00	40.00	41.00	22.00	22.00
Yes	4.00	4.00	4.00	4.00	66.00	66.00	25.00	25.00
Yes	2.00	2.00	3.00	3.50	6.00	6.00	6.00	9.00
Yes	4.00	4.00	4.00	4.00	63.00	63.00	31.00	31.00
Yes	4.00	4.00	4.00	4.00	64.00	64.00	31.00	31.00
No	4.00	4.00	4.00	4.00	64.00	66.00	32.00	34.00
No	4.00	4.00	4.00	4.00	62.00	62.00	33.00	33.00
No	4.00	4.00	4.00	4.00	66.00	66.00	33.00	33.00
No	4.00	4.00	4.00	4.00	63.00	63.00	26.00	26.00
No	4.00	4.00	4.00	4.00	66.00	66.00	30.00	30.00
No	4.00	4.00	4.00	4.00	66.00	66.00	32.00	32.00
No	4.00	4.00	4.00	4.00	38.00	59.00	28.00	29.00
Yes, summary (n=8)	*3.50 [2.75–4.00]*	*3.50 [2.75–4.00]*	*4.00 [3.00–4.00]*	*4.00 [3.38–4.00]*	*51.50 [28.75–63.25]*	*51.50 [29.50–63.25]*	*23.50 [20.25–28.75]*	*23.50 [20.25–31.00]*
No, summary (n=7)	*4.00 [4.00–4.00]*	*4.00 [4.00–4.00]*	*4.00 [4.00–4.00]*	*4.00 [4.00–4.00]*	*64.00 [62.50–66.00]*	*66.00 [62.50–66.00]*	*32.00 [29.00–32.50]*	*32.00 [29.50–33.00]*

MAS, Modified Ashworth Scale (lower limb); MMT, manual muscle test; FMA, Fugl-Meyer Assessment. Subgroup definitions as in Table S1. Individual rows list each patient's value before and after treatment; summary rows report median [IQR] for each subgroup, consistent with Table 3 for the full cohort. Because acupotomy was administered according to baseline spasticity severity (MAS ≥ 2), the subgroups differed systematically at baseline and were not directly comparable. Therefore, these data are presented for descriptive purposes only and should not be interpreted as evidence of the independent effect of acupotomy; no formal between-group statistical testing was performed.

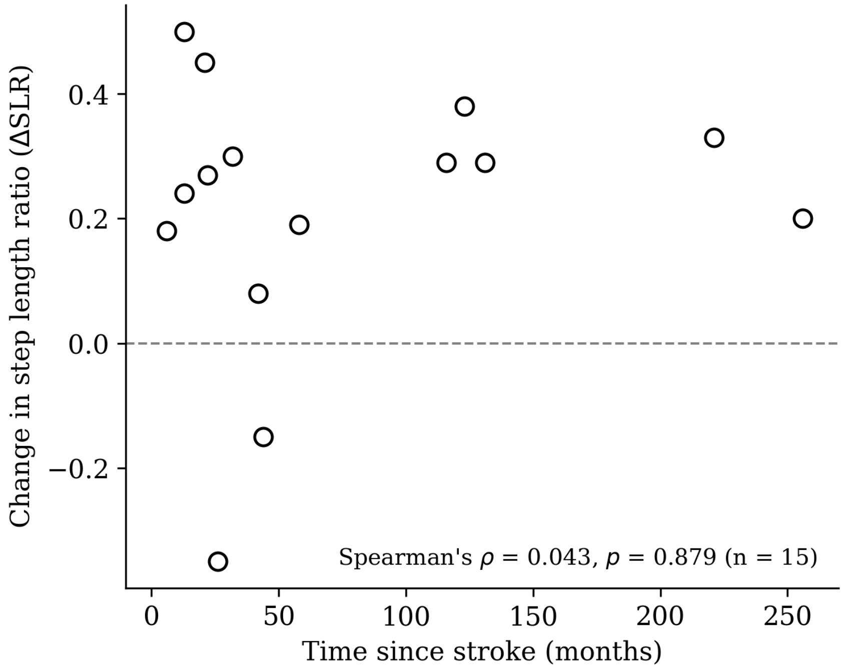

Figure S1. Association between time since stroke and change in step length ratio (ΔSLR).

ΔSLR was calculated as the post-treatment step length ratio minus the pre-treatment step length ratio for each patient (n = 15). An exploratory Spearman correlation analysis (see Methods, Section 2.5) showed no statistically significant association between time since stroke and ΔSLR (Spearman's $\varrho = 0.043$, $p = 0.879$). Given the small sample size and the wide, right-skewed distribution of time since stroke (6–256 months), this analysis was underpowered, and the near-zero correlation coefficient should not be interpreted as definitive evidence of no association.
